# Systemic Therapy for Advanced Soft Tissue Sarcomas

**DOI:** 10.1002/cncr.26415

**Published:** 2011-08-11

**Authors:** Richard F Riedel

**Affiliations:** Division of Medical Oncology, Duke University Medical CenterDurham, North Carolina

**Keywords:** soft tissue sarcoma, novel therapies, mammalian target of rapamycin (mTOR) inhibitors, tyrosine kinase inhibitors, maintenance therapy

## Abstract

Soft tissue sarcomas (STS) are a rare, heterogeneous group of solid tumors in need of improved therapeutic options. First-line chemotherapy is considered the current standard of care for patients with advanced, symptomatic STS, but the median survival is only 8 to 12 months. Efforts to increase response rates by using combination or dose-dense regimens have largely failed to improve patient outcomes. However, increasing evidence supports the use of specific treatments for certain histological subtypes of STS, and novel therapies, including tyrosine kinase and mammalian target of rapamycin inhibitors, are currently under active investigation. In addition, novel treatment approaches (such as maintenance therapy) designed to prolong the duration of response to chemotherapy and delay disease progression are being explored. This article provides an overview of current systemic therapies for patients with advanced STS and discusses ongoing efforts designed to improve patient outcomes through the use of novel therapeutic agents and treatment strategies. Cancer 2011;. © 2011 American Cancer Society.

Soft tissue sarcomas (STS) are a rare, heterogeneous group of solid tumors in need of improved therapeutic options. This article provides an overview of current systemic therapies for patients with advanced STS and discusses ongoing efforts designed to improve patient outcomes through the use of novel therapeutic agents and treatment strategies.

## INTRODUCTION

Soft tissue sarcomas (STS) are a heterogeneous group of solid tumors of mesenchymal origin that account for < 1% of all adult and 15% of pediatric malignancies in the United States. These tumors can arise anywhere in the body, but develop predominantly in the limbs, limb girdle, and abdominal cavity.^1^ Overall, an estimated 10,520 new STS cases are expected to be diagnosed in 2010 in the United States, and 3920 individuals will die of these tumors.^2^ The overall 5-year survival rate for patients with STS is approximately 50% to 60% in adults and 75% in children, depending on tumor grade, size, depth, site, and histological subtype.^3^-^5^

Surgical resection, with the goal of achieving an appropriately negative margin, is the primary modality of treatment and is considered definitive for patients with localized low-grade tumors.^6^ Adjuvant radiation with or without chemotherapy is indicated for patients with high-grade STS [stage II-III; American Joint Committee on Cancer, Cancer Staging Manual, Seventh Edition (2010)]. Alternatively, these modalities may be delivered preoperatively to reduce tumor size or improve resectability, particularly in potentially resectable cases or when there are concerns for adverse functional outcomes after surgery.^6^ Patients with stage IV disease are characterized as having regional lymph node involvement or distant metastases, regardless of tumor size or depth.^1^ Patients with advanced local disease or metastatic disease have a particularly poor prognosis, with a median overall survival (OS) of only 8 to 12 months.^7^ However, outcome varies depending on the histologic subtype (eg, metastatic disease may persist for ≥ 10 years in patients with alveolar soft part sarcoma [ASPS]).^7^-^10^

The treatment of stage IV disease depends on the extent of metastases. The lung is the most common site of metastasis in patients with STS of the extremities, whereas extrapulmonary metastases usually develop as a later manifestation of widely disseminated disease.^9^ Liver metastases are also frequently encountered in patients with STS in the abdominal cavity.^9^ Patients with limited metastases confined to a single organ or regional lymph node may be considered for resection whenever possible and subsequently managed in the same manner as those with high-grade disease.^6^, ^11^ Patients with disseminated metastases, however, should be considered for palliative systemic therapy.^11^, ^12^ This article provides an overview of the current approach to systemic therapy in patients with unresectable and metastatic STS (excluding gastrointestinal stromal tumors [GIST]), highlights novel therapies, and introduces new treatment paradigms that may offer the potential to improve outcomes.

### Current Approach to the Treatment of Patients With Advanced STS

Current guidelines from the National Comprehensive Cancer Network (NCCN) and the European Society for Medical Oncology (ESMO) consider single-agent or combination regimens as options for the first-line treatment of patients with advanced unresectable STS.^6^, ^11^ Chemotherapy may be continued until disease progression, although clinical efficacy decreases over time and cumulative toxicity may become problematic as additional cycles are administered (eg, higher cumulative doses of doxorubicin cause cardiomyopathy and an associated mortality risk).^13^, ^14^ The guidelines also support a “watchful waiting” approach for asymptomatic patients diagnosed with advanced disease, especially those patients with long disease-free intervals and minimal disease burden and patients who have achieved best response with first-line chemotherapy.^6^, ^12^ The lack of evidence supporting the continuation of chemotherapy until disease progression, the limitations of the watchful waiting approach (including potential disease progression and associated patient anxiety), and the poor OS of patients with advanced STS underscore the need for new therapies and treatment approaches.

In first-line chemotherapy, efforts were made to improve the objective response rate (ORR), either by increasing the dose intensity or administering active agents in combination, with the goal of improving patient survival.^15^ However, although some improvement was achieved, it did not translate into a survival benefit. A pooled analysis of 8 randomized clinical trials involving 2281 patients with STS demonstrated a slight trend favoring doxorubicin-based combination therapy for ORR (odds ratio [OR], 0.79; 95% confidence interval [95% CI], 0.60-1.05 [*P* = .10]); these results, as well as OS data (OR, 0.84; 95% CI, 0.67-1.06 [*P* = .13]), did not reach statistical significance compared with single-agent doxorubicin.^16^ Nausea, vomiting, and myelosuppression were consistently more severe with the combination regimens.

The European Organisation for Research and Treatment of Cancer (EORTC) Soft Tissue and Bone Sarcoma Group retrospectively evaluated factors important in predicting response and survival among 2185 patients with advanced STS who received a first-line anthracycline-containing regimen.^8^ For the entire cohort, the ORR was 26% and the median OS was 51 weeks. Although the absence of liver metastases and younger age of the patients were found to be independently associated with both response and survival, high histopathological grade was associated with response to chemotherapy, whereas low histopathological grade was associated with survival, suggesting that the ORR may not be sufficient for determining the potential clinical benefit of new agents for the treatment of STS.

#### Single-agent regimens

Single-agent chemotherapy with doxorubicin, ifosfamide, or dacarbazine and combination regimens with or without an anthracycline backbone have been widely used to treat patients with disseminated metastatic STS (Table 1).^15^-^29^ Doxorubicin is the single most active agent in the treatment of metastatic STS, producing ORRs of 16% to 27% in clinical trials.^16^, ^17^ Although the response to doxorubicin may depend on dose intensity, this needs to be balanced against the greater toxicity associated with higher doses (eg, cardiotoxicity).^30^

**Table 1:** Options for First-Line Chemotherapy in Patients With Advanced STS

Treatment	Response Rate	Median OS, Months	Study
Single-agent regimen			
Doxorubicin (60-75 mg/m^2^ every 3 wk)	16%-27%	7.7-12.0	Bramwell 2000^16^
Lorigan 2007^21^			
Epirubicin (75 mg/m^2^)	18%	4.0	Mouridsen 1987^18^
Ifosfamide (5 g/m^2^ over 24 h every 3 wk)	10%-25%	12.0	van Oosterom 2002^19^
High-dose ifosfamide^a^	25%-38%	10.2-18.5	van Oosterom 2002^19^
Buesa 1998^20^			
Temozolomide			
(Oral bid × 5 d every 4 wk)^b^	8%	13.2	Talbot 2003^15^
(Oral every d × 6 wk; then 3 wk off treatment)^b^	16%	8.1	Garcia del Muro 2005^22^
Dacarbazine (1.2 g/m^2^ every 3 wk)	18%	NR	Buesa 1991^29^
Combination regimens			
Doxorubicin (50 mg/m^2^) + ifosfamide (5 g/m^2^) every 3 wk	21%-28%	13.8-14.0	Santoro 1995^24^
Le Cesne 2000^28^			
Doxorubicin (60 mg/m^2^) + ifosfamide (7.5 g/m^2^ over 2 d) every 3 wk	34%	∼11.5	Edmonson 1993^23^
Doxorubicin (60 mg/m^2^) + dacarbazine^c^	17%-30%	8.0-12.0	Borden 1987^17^
Antman 1993^25^			
Mesna, doxorubicin, ifosfamide, and dacarbazine (MAID)^d^	32%	13.0	Antman 1993^25^
Gemcitabine (900 mg/m^2^ on d 1 and 8) + docetaxel (100 mg/m^2^) on d 8 every 3 wk	16%	17.9	Maki 2007^26^
Gemcitabine (800 mg/m^2^) + vinorelbine (25 mg/m^2^) on d 1 and 15 every 4 wk	13%	75% (12-mo OS)	Dileo 2007^27^

Abbreviations: bid, twice daily; NR, not reported; OS, overall survival; STS, soft tissue sarcoma.

a High-dose ifosfamide regimens included 9 g/m^2^ over 3 days every 3 weeks or 14 g/m^2^ over 6 days every 4 weeks.

b Temozolomide was administered orally at a loading dose of 200 g/m^2^, then every 12 hours at 90 mg/m^2^ for 4.5 days every 4 weeks, or it was administered at doses of 75 mg/m^2^ or 100 mg/m^2^ once daily for 6 weeks followed by a 3-week treatment break before the next cycle.

c Dacarbazine was administered intravenously every 3 weeks at a dose of 250 mg/m^2^/day every 5 days or 1000 mg/m^2^/day every 4 days.

d MAID was comprised of doxorubicin at a dose of 60 mg/m^2^ and dacarbazine at a dose of 1000 mg/m^2^ infused continuously over 4 days plus ifosfamide at a dose of 7.5 g/m^2^ and mesna at a dose of 10 g/m^2^ infused continuously over 3 or 4 days. The ifosfamide dose was subsequently reduced to 6 g/m^2^ due to unacceptable myelosuppression after 154 of 374 patients had been accrued.

Epirubicin and the liposomal anthracyclines were developed to improve the safety profile of doxorubicin. Epirubicin tended to be slightly less efficacious than doxorubicin (ORR, 18% vs 25%; *P* = .33), but produced less hematological toxicity and less nausea and vomiting.^18^ Improved ORRs were reported with higher doses of epirubicin at the expense of greater toxicity.^31^ However, in a cohort of 334 patients with advanced STS, 2 different schedules of high-dose epirubicin failed to improve the ORR or OS when compared with a standard dose of doxorubicin (75 mg/m^2^), and any toxicity advantage was lost.^32^ Pegylated liposomal doxorubicin appeared to be as effective as standard-dose doxorubicin in a randomized trial of patients with advanced STS (N = 94).^33^ However, in this study, both agents produced low ORRs (10% and 9%, respectively), but had differing toxicity profiles. In other phase 2 trials, ORRs with pegylated liposomal doxorubicin ranged from 0% to 10%, although approximately one-third of the patients achieved stable disease (SD).^15^, ^34^, ^35^

Standard-dose ifosfamide is active in the first-line treatment of patients with advanced STS (ORRs of 10%-25%).^19^, ^36^ High-dose ifosfamide (HDI) regimens produced ORRs as high as 38%, but were associated with higher hematologic and nonhematologic toxicities than the standard dose.^19^, ^20^, ^37^ The EORTC Soft Tissue and Bone Sarcoma Group compared 2 investigational HDI schedules versus standard-dose doxorubicin in a phase 3 trial of patients with advanced STS (N = 326).^21^ No differences in ORR, progression-free survival (PFS), or OS were observed, but myelosuppression occurred more frequently with HDI. Higher doses may be effective in patients who develop disease progression or recurrence after doxorubicin pretreatment and/or first-line standard-dose ifosfamide.^38^ In a phase 2 study of patients whose disease had progressed after pretreatment, HDI produced responses in 33% of patients and SD in 22%. It is interesting to note that 24% of patients with disease refractory to standard-dose ifosfamide achieved partial responses (PR); the median duration of response was 8 months and the median OS was 12 months. However, HDI was associated with dose-limiting neutropenia, as well as neurotoxicity and renal toxicity. In a subsequent EORTC multicenter phase 2 trial, HDI administered with adequate mesna protection appeared to be somewhat less effective.^39^

Dacarbazine has been available for more than 3 decades. In a pooled analysis of published and unpublished data, the ORR of single-agent dacarbazine was 18%.^40^ In a phase 2 trial of patients with metastatic STS (N = 11), temozolomide, an oral prodrug of dacarbazine, produced an ORR of 8%.^15^ The ORR rate improved to 16% when temozolomide was administered once daily for 6 weeks followed by a 3-week break from treatment in a patient population with pretreated STS.^22^

#### Combination chemotherapy regimens

Although combination regimens involving anthracyclines, ifosfamide, and dacarbazine were developed to increase ORRs and improve patient outcomes, studies with these regimens were largely unsuccessful at improving outcomes, often increasing the toxicity burden. The Eastern Cooperative Oncology Group (ECOG) conducted a phase 3 trial comparing the combination of ifosfamide and doxorubicin with single-agent doxorubicin in patients with metastatic STS (N = 178).^23^ Although the combination produced a significantly higher ORR (34% vs 20%; *P* = .03), it was associated with greater myelosuppression and did not significantly prolong OS. This study also included a third treatment arm with mitomycin, doxorubicin, and cisplatin, which produced a higher ORR than doxorubicin without increasing myelotoxicity; again, survival was not improved. In an EORTC phase 3 trial, the combination of ifosfamide and doxorubicin did not significantly improve the ORR (28% vs 23%), median response duration (44 weeks vs 46 weeks), or median OS (55 weeks vs 52 weeks) compared with single-agent doxorubicin, but did increase hematologic toxicity.^24^ EORTC-62012 is an ongoing phase 3 trial comparing the combination of doxorubicin and ifosfamide (with pegfilgrastim support) versus doxorubicin alone.

The combination of doxorubicin and dacarbazine has a response rate that is comparable to or better than single-agent doxorubicin (Table 1).^16^, ^17^, ^21^, ^25^ An intensified regimen comprised of mesna, doxorubicin, ifosfamide, and dacarbazine (MAID) significantly increased the ORR (32% vs 17%; *P* < .005) and prolonged time to disease progression (TTP) (6 months vs 4 months; *P* < .02) compared with the combination of doxorubicin and dacarbazine in a phase 3 trial.^25^ However, MAID did not improve OS and was associated with greater myelosuppression.

Several other drugs, including gemcitabine, docetaxel, and vinorelbine, have been evaluated in combination regimens in phase 2 trials, largely involving patients with disease that is resistant to front-line chemotherapy. Compared with gemcitabine alone, the gemcitabine and docetaxel combination produced a higher ORR (16% vs 8%) and longer median OS (17.9 months vs 11.5 months; *P* = .03), albeit with greater toxicity, in patients with metastatic STS (N = 122).^26^ However, a phase 2, retrospective, pooled analysis of patients with leiomyosarcoma (N = 121) who were treated with the combination of gemcitabine and docetaxel in the second-line (n = 84) setting demonstrated no significant improvement in the ORR or median PFS relative to single-agent gemcitabine.^41^ A gemcitabine and vinorelbine combination produced clinical benefit (25%) in a single-arm study and was associated with a 12-month OS rate of 75%.^27^

Several studies have explored dose-escalated doxorubicin and ifosfamide combination regimens with colony-stimulating factor (CSF) support. The EORTC compared standard and intensified doses of doxorubicin in combination with ifosfamide in a phase 3 trial of patients with advanced STS (N = 314).^28^ Granulocyte-macrophage–CSF (GM–CSF) was administered to patients receiving the intensified dose. The intensified regimen significantly prolonged the median TTP compared with the standard-dose regimen (29 weeks vs 19 weeks), but did not improve OS. Similarly, an intensified MAID regimen with granulocyte (G-CSF) support, in which the doses of doxorubicin, ifosfamide, and dacarbazine were 20% to 33% higher than those in the standard MAID regimen, failed to improve the ORR, PFS, or OS in a recent phase 3 trial.^42^ High-dose chemotherapy followed by peripheral blood stem cell transplantation did not improve the outcomes of patients who responded to first-line MAID.^43^ Because these regimens do not significantly improve patient outcomes, better treatment options are still needed.

### Treatment Considerations for Certain Sarcoma Subtypes

For many clinical trials evaluating chemotherapeutic agents, patients with advanced STS have been grouped together, regardless of tumor histology. However, growing evidence suggests that for patients with certain histological subtypes, specific agents besides doxorubicin may be preferred options.

#### Angiosarcoma

Current NCCN guidelines recommend paclitaxel and bevacizumab as treatment options for patients with angiosarcoma, a rare STS subtype with an aggressive clinical course.^6^ In a phase 2 trial (N = 26), bevacizumab produced a 12% PR rate in patients with angiosarcoma and epithelioid hemangioendothelioma.^44^ Paclitaxel has exhibited low activity in nonselected sarcoma populations, but favorable responses in patients with angiosarcomas of the scalp.^45^, ^46^ Paclitaxel is highly active in angiosarcomas, with an ORR of 62% and a median TTP of 7.6 months.^47^ In a phase 2 trial examining patients with advanced angiosarcomas (N = 27), paclitaxel demonstrated a 19% PR rate after 2 cycles, a median TTP of 4 months, and a median OS of 8 months, but no difference in PFS was noted between chemotherapy-treated and chemotherapy-naive patients.^48^

Other nonapproved treatment options for angiosarcomas include multikinase inhibitors (eg, sorafenib and sunitinib) and liposomal doxorubicin. In phase 2 trials, sorafenib produced a 14% PR rate and a median OS of 14.3 months in an angiosarcoma trial (N = 37),^49^ whereas sunitinib did not produce a response rate in patients with angiosarcoma (n = 2) or fibrous tumor/hemangiopericytoma (n = 3).^50^ In another phase 2 trial, pegylated liposomal doxorubicin demonstrated similar efficacy and reduced myelosuppression compared with doxorubicin in patients with advanced STS.^33^ Other results suggest that liposomal doxorubicin might be considered as a palliative therapy in patients with cutaneous angiosarcoma.^51^

#### Leiomyosarcoma

Leiomyosarcomas are generally believed to be insensitive to conventional chemotherapy. A phase 2 trial has shown that the combination of gemcitabine and docetaxel has some activity in leiomyosarcomas.^52^ Patients with unresectable leiomyosarcomas (N = 34), mostly of the uterine type, received the combination of gemcitabine and docetaxel; 16 of the patients were treated after developing disease progression while receiving doxorubicin-based therapy. This regimen produced a 53% response rate and a median TTP of 5.6 months. The same regimen was evaluated as second-line treatment for patients with advanced uterine leiomyosarcomas; the ORR (27%) was lower than in the first trial, but an additional 50% of the patients had SD for a median of 5.4 months and the 24-week PFS rate was 52%.^53^ Although a recent pooled analysis suggests the combination of gemcitabine and docetaxel has only limited benefit over gemcitabine alone,^41^ other reports have demonstrated the activity of the gemcitabine and docetaxel combination in leiomyosarcomas and also suggest that this regimen is active in other sarcoma subtypes.^26^, ^54^

#### Synovial sarcoma

In synovial sarcomas, both the size and location of the primary tumor are independent factors that govern disease-free survival after surgical resection of localized disease.^55^, ^56^ For adults with high-risk synovial sarcomas, neoadjuvant/adjuvant ifosfamide-based therapy has been associated with improved disease-specific survival.^55^ In a phase 3 ECOG trial, the combination of ifosfamide and doxorubicin produced a significantly higher ORR compared with single-agent doxorubicin (88% vs 20%; *P* = .02) in a small subgroup of patients with advanced synovial sarcoma (N = 20),^23^ whereas a PR rate of 42% and a median OS of 11 months were observed in a follow-up study.^57^ In a retrospective analysis of patients with advanced STS (N = 1337) who received first-line ifosfamide-based therapy, synovial sarcoma histology was identified as an independent, favorable prognostic factor for both treatment response and OS.^58^

The DNA-binding cytotoxic agent trabectedin (ecteinascidin-743) and the multikinase inhibitor pazopanib may also have antitumor activity in synovial sarcoma. In a retrospective analysis of patients (N = 39) with advanced/metastatic disease who were treated with trabectedin, 18% achieved a PR, 5% had minor responses, and 28% experienced SD.^59^ In a phase 2 study of pazopanib in patients (N = 37) with recurrent/refractory advanced STS, 13% of patients with synovial sarcoma experienced a PR, with 49% remaining free of disease progression at 12 weeks.^60^

### Novel Therapeutics

#### Cytotoxic agents

Trabectedin is approved in Europe and 25 other countries (excluding the United States) for the treatment of patients with advanced STS after failure of an anthracycline and ifosfamide and in patients who are not suitable candidates for these agents.^6^, ^61^-^63^ In an EORTC phase 2 trial of 99 patients, a PR rate of 8% and an SD rate of 26% (of > 6 months) were observed in patients treated with trabectedin who failed previous chemotherapy regimens.^64^ The median PFS was 105 days and the median OS was 9.2 months. Trabectedin was generally well tolerated, with neutropenia and asymptomatic transaminase elevations reported to be the main toxicities. Comparable results were reported in other phase 2 trials involving more heavily pretreated patients.^65^, ^66^ Trabectedin has also shown promising activity in a retrospective analysis of pretreated patients (N = 51) with advanced myxoid liposarcomas (ORR of 51% and a median PFS of 14 months).^67^ Currently, trabectedin is being evaluated against dacarbazine in a randomized, multicenter, phase 3 trial in patients with advanced pretreated L-sarcoma (liposarcoma or leiomyosarcoma).^68^

TH-302, a hypoxia-activated cytotoxic prodrug, was administered in combination with doxorubicin and produced a 25% PR rate in a phase 1/2 trial of patients with advanced or metastatic STS (N = 20).^69^ Palifosfamide, the active moiety of the chemotherapy prodrug ifosfamide, in combination with doxorubicin, was found to double the PFS over doxorubicin alone (7.8 months vs 4.4 months) in a phase 2 trial of patients with unresectable or metastatic STS.^70^ An ongoing phase 3 trial is examining this combination as a front-line treatment for patients with STS.^71^

#### Tyrosine kinase inhibitors

To our knowledge, no tyrosine kinase inhibitors (TKIs) have been approved to date for the treatment of sarcoma other than GIST. Pazopanib, which targets vascular endothelial growth factor receptor (VEGFR) and platelet-derived growth factor receptor (PDGFR), has shown promising activity in patients with advanced STS.^60^ Compared with historical controls receiving second-line chemotherapy, pazopanib prolonged PFS and OS in patients with STS (including leiomyosarcomas and synovial sarcomas). Pazopanib was generally well tolerated; the most common grade 3/4 toxicities were hypertension, fatigue, and hyperbilirubinemia. Based on these findings, the EORTC initiated the *Pa*zopanib Exp*l*or*e*d in Sof*t T*issue Sarcoma a Phas*e* 3 (PALETTE) trial to compare pazopanib with placebo in patients (N = 369) whose disease had progressed during or after at least 1 anthracycline-containing regimen. Preliminary results have indicated that although treatment with pazopanib did not improve OS, the median PFS was significantly prolonged by 13 weeks.^72^

Other multikinase inhibitors have been tested in patients with advanced STS, generally with limited success reported to date. In a phase 2 trial (N = 48), sunitinib produced a PR in a patient with a desmoplastic round cell tumor and SD lasting ≥ 16 weeks in 10 additional patients, including 2 of 3 patients with solitary fibrous tumors. Patients who underwent positron emission tomography/computed tomography scanning (n = 21) demonstrated evidence of a metabolic PR or SD.^50^ Sorafenib has exhibited single-agent activity against angiosarcomas, but showed minimal activity against other histological subtypes.^49^ Skin toxicity was the dose-limiting side effect. Sorafenib has also demonstrated activity in desmoid tumors, which are fibroblastic neoplasms that arise from musculoaponeurotic stromal elements.^73^ Imatinib, a KIT/PDGFR/BCR-ABL TKI that is used as first-line therapy for patients with unresectable/metastatic GIST, was evaluated in 10 histological sarcoma subtypes, producing responses in 4 of 185 patients (2%).^74^ When SD (> 4 months) was included, the clinical benefit rate (CBR) was 15% overall, with the highest CBR noted in patients with liposarcomas and leiomyosarcomas.

ARQ 197, a selective inhibitor of the c-Met receptor tyrosine kinase, is indicated for a rare group of tumors associated with the microphthalmia transcription factor family, including clear cell sarcoma (CCS) and ASPS.^75^, ^76^ In a phase 2 trial of patients with CCS (n = 7), ASPS (n = 17), and translocation-associated renal cell carcinoma, treatment with ARQ 197 demonstrated a 14% PR rate in patients with CCS and a SD rate of 29% and 59%, respectively, in patients with CCS and ASPS. Treatment with the fibroblast growth factor receptor and VEGFR inhibitor brivanib in patients with advanced STS has been shown to prolong PFS (from week 12) in comparison with placebo (2.8 months vs 1.4 months) in basic fibroblast growth factor (FGF2)-positive patients.^77^ Cediranib, another VEGFR inhibitor, has also demonstrated promising preliminary results; in 1 study (N = 7), 4 PRs and 1 case of SD^78^ were observed and in another study (N = 36), a 43% PR rate was noted in evaluable patients (n = 28).^79^ Axitinib, a small-molecule TKI with multiple targets, is currently being investigated in patients with advanced/metastatic STS.^80^

#### Insulin-like growth factor 1 inhibitors

Several anti–insulin-like growth factor 1 monoclonal antibodies are being investigated in patients with advanced sarcoma, including figitumumab and cixutumumab (IMC-A12). Figitumumab has been evaluated in a phase 1 study in patients with sarcoma and Ewing sarcoma; among patients with STS (N = 9), an SD rate of 56% and a 44% progressive disease rate were observed.^81^ Figitumumab has also been investigated in combination with everolimus in a phase 1 study of patients with advanced sarcoma and other advanced tumors (N = 21); of 18 evaluable patients, 1 achieved a PR and 83% experienced SD.^82^ In a phase 2 trial (N = 113), initial results with single-agent cixutumumab demonstrated the highest CBR (57%) in patients with adipocytic sarcoma (n = 37).^83^ Currently, cixutumumab is being investigated in combination with temsirolimus in patients with metastatic sarcomas.^84^

#### Mammalian target of rapamycin inhibitors

The rationale for mammalian target of rapamycin (mTOR) inhibitors in patients with advanced STS has been reviewed.^85^, ^86^ In a case study, treatment with sirolimus (rapamycin, the prototype mTOR inhibitor) and cyclophosphamide produced a PR in a patient with metastatic myxoid chondrosarcoma, a slow-growing sarcoma that responds poorly to chemotherapy and radiotherapy.^87^ Treatment with sirolimus in patients (n = 3) with perivascular epithelioid cell tumors also resulted in radiographic responses as well as molecular tuberous sclerosis complex (TSC) and mTOR complex changes.^88^ In a phase 2 trial, treatment with everolimus in patients (N = 61) with recurrent or refractory sarcoma produced a complete response (CR), PR, or SD (after 4 months) in 13% of patients with STS or bone sarcoma and 27% of patients with GIST.^89^ Common adverse events (AEs) included mucositis, fatigue, and elevation of liver transaminases. In combination with imatinib, everolimus has demonstrated activity in a phase 1/2 study of imatinib-resistant GIST.^90^ Everolimus also reduces tumor volume by regulating gene products of the TSC in subependymal giant cell astrocytomas.^91^ Temsirolimus was investigated in a phase 2 trial of patients (N = 40) with advanced sarcoma, but only 5% of evaluable patients (those with undifferentiated fibrosarcoma and uterine leiomyosarcoma) achieved a PR.^92^ Tolerability was poor, with 43% of evaluable patients experiencing an AE of grade 3 or higher possibly related to the drug. Ridaforolimus has demonstrated antitumor activity in 2 phase 1 trials that included patients with advanced STS.^93^, ^94^ In a subsequent phase 2 trial of patients (N = 212) with advanced STS or bone sarcomas, the majority of whom were previously treated, treatment with ridaforolimus resulted in a 29% CBR and a median OS of 40 weeks.^95^ The most common AEs were fatigue, stomatitis, hypertriglyceridemia, anemia, rash, and nausea. In a phase 1 trial, an oral formulation of ridaforolimus produced a 23% CBR in the subgroup of patients with advanced sarcomas.^96^

#### Other agents

In phase 1 and 2 trials, the Akt inhibitor perifosine has been evaluated in patients with advanced STS. One study failed to meet the primary objective of a 40% PFS rate at 6 months,^97^ whereas in another study, no objective responses were observed and 27% of patients experienced SD.^98^ In contrast, a retrospective analysis (N = 60) demonstrated a 5% PR rate in evaluable patients (45% of patients with SD for ≥ 4 months),^99^ suggesting that perifosine may not be particularly effective in producing tumor responses but may have some role in disease stabilization. The mitotic inhibitor eribulin was examined in an EORTC phase 2 trial of patients with STS stratified by subtype and met prespecified statistical boundaries for the primary endpoint of PFS at 12 weeks for both leiomyosarcoma and adipocytic sarcoma.^100^ Eribulin is currently being evaluated in a phase 3 clinical trial for patients with advanced, refractory STS.^101^ ABT-510, a peptide that mimics the antiangiogenic activity of thrombospondin-1, was tested in a phase 2 trial in patients (N = 88) with locally advanced or metastatic STS. Approximately 50% of patients achieved SD, and only 1 objective response occurred.^102^ Rexin-G (Epeius Biotechnologies, San Marino, Calif), a gene therapy vector bearing a cytocidal dominant negative cyclin D1 construct, was investigated in a phase 1/2 study of patients with STS and bone sarcomas (N = 20); 65% of patients achieved SD according to Response Evaluation Criteria In Solid Tumors (RECIST) criteria.^103^ Histone deacetylase (HDAC) inhibitors have also shown some promise in treating sarcoma. A phase 1/2 trial of patients with metastatic sarcoma is currently examining the safety and tolerability of the HDAC inhibitor valproic acid in combination with bevacizumab, gemcitabine, and docetaxel.^104^

### Maintenance Therapy as a New Strategy for the Treatment of Sarcoma

**Figure 1:**
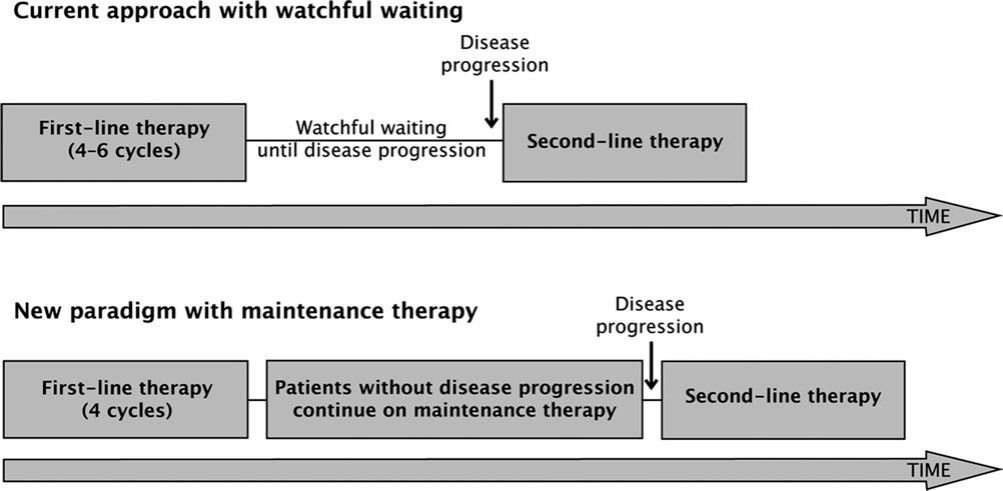
Elements of “watchful waiting” versus maintenance treatment are shown. Watchful waiting indicates first-line chemotherapy followed by monitoring until disease progression; maintenance treatment, first-line chemotherapy followed by maintenance therapy until disease progression.

Maintenance therapy has been developed as part of treatment paradigms to prolong response duration and delay disease progression in responsive patients or patients with SD after a defined number of chemotherapy cycles (Fig. .1). Given the inherent toxicity associated with first-line chemotherapy and the need to continue maintenance therapy for prolonged periods of time, agents used in maintenance therapy should be well tolerated. Some chemotherapeutic agents, as well as targeted therapies with cytostatic properties and documented tolerability, have been found to be effective as maintenance therapy in patients with non–small cell lung cancer and ovarian cancer.^105^-^107^ However, some studies have not demonstrated a benefit of maintenance therapy.^108^

**Figure 2:**
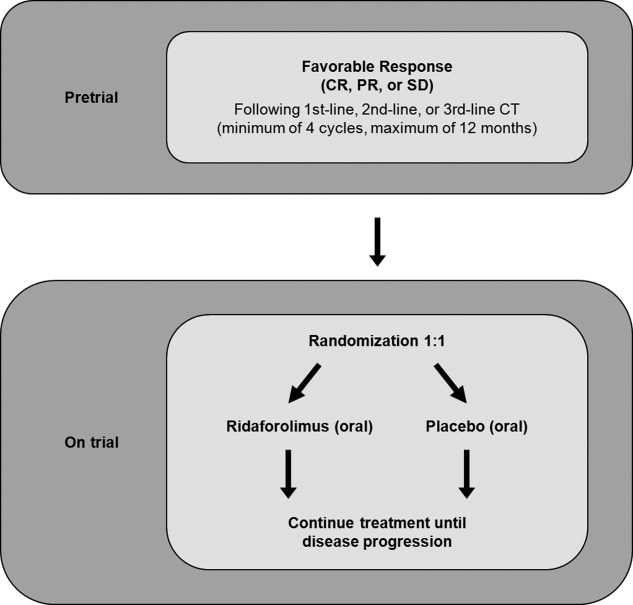
The *S*arcoma M*u*lti-*c*enter *C*linical *E*valuation of the *E*fficacy of Ri*d*aforolimus (SUCCEED) study scheme is shown. CR indicates complete response; PR, partial response; SD, stable disease; CT, chemotherapy.

The *S*arcoma M*u*lti-*c*enter *C*linical *E*valuation of the *E*fficacy of Ri*d*aforolimus (SUCCEED) trial, one of the largest studies of patients with metastatic STS or bone sarcoma published to date, is a pivotal phase 3 trial that evaluated maintenance therapy with oral ridaforolimus in patients (N = 711) who achieved a favorable response (CR, PR, or SD) after receiving a minimum of 4 cycles of chemotherapy (Fig. .2). Preliminary data have demonstrated a significant increase in PFS (ridaforolimus vs placebo), with a 21% improvement in the median PFS (17.7 weeks vs 14.6 weeks; hazard ratio [HR], 0.72 [*P* = .0001]) and a nonstatistically significant trend toward an OS benefit (21.4 months vs 19.2 months; HR, 0.88 [*P* = .2256]).^109^ The incidence of stomatitis and other AEs was higher in patients receiving ridaforolimus, and the overall safety profile was considered to be similar to that of other mTOR inhibitors. Further studies will help confirm the benefit of maintenance therapy with mTOR inhibitors in patients with advanced STS.

### Conclusions

The current standard of care for patients with unresectable advanced STS includes first-line treatment with single-agent doxorubicin or a doxorubicin-based combination chemotherapy regimen. However, patients in this setting have a poor prognosis. Efforts to increase ORRs through the use of combination or dose-intense regimens have had little impact on patient outcome. Several recent advances offer promise for the treatment of patients with advanced STS. First, increasing evidence suggests that patients with certain STS histologies may benefit from specific treatments, such as paclitaxel or sorafenib in those with angiosarcomas and ifosfamide in patients with synovial sarcomas. Second, several targeted drugs have demonstrated clinical benefit in patients with advanced STS, and ongoing efforts are exploring how to best deploy such agents in conjunction with existing treatment options. Finally, drawing on clinical experience in treating other malignancies, maintenance therapy is being evaluated as a new treatment paradigm for patients with advanced disease. These data suggest that the future is bright for improving treatment options and outcomes for patients with advanced STS.

## FUNDING SUPPORT

Editorial assistance was funded by Merck & Co., Inc. Dr. Riedel was responsible for all content and editorial decisions and received no compensation for the development of the article.

## CONFLICT OF INTEREST DISCLOSURES

Dr. Riedel has received clinical research support from Novartis Pharmaceuticals, ARIAD Pharmaceuticals, and Ziopharm Oncology and an honorarium from ARIAD Pharmaceuticals. He is also a consultant for Merck & Co. Inc, and has served on speakers bureaus for both Novartis and Pfizer, Inc.
